# Observed Differences in Patient Comorbidities and Complications Undergoing Primary Total Joint Arthroplasty Between Non-orthopaedic and Orthopaedic Referral Patients

**DOI:** 10.7759/cureus.59258

**Published:** 2024-04-29

**Authors:** Bennett W Feuchtenberger, Michael C Marinier, Kyle Geiger, Matthew Van Engen, Natalie A Glass, Jacob Elkins

**Affiliations:** 1 Department of Orthopaedic Surgery, University of Iowa Carver College of Medicine, Iowa City, USA; 2 Department of Orthopaedic Surgery, University of Iowa Hospitals and Clinics, Iowa City, USA; 3 Department of Orthopaedic Surgery, University of Iowa, Iowa City, USA; 4 Department of Orthopaedic Surgery, University of Iowa Hospitals and Clinics/University of Iowa Carver College of Medicine, Iowa City, USA

**Keywords:** bundled payment for care improvement, reimbursement, referral status, complications, comorbidity, total joint arthroplasty

## Abstract

Background: Value-based total joint arthroplasty (TJA) has resulted in decreasing surgeon reimbursement which has created concern that surgeons are being incentivized to avoid medically complex patients. The purpose of this study was to determine if patients who underwent primary total knee (TKA) and total hip arthroplasty (THA) had different comorbidities and complication rates based on referral type: 1) non-orthopaedic referral (NOR), 2) outside orthopaedic referral (OOR) or 3) self-referral (SR).

Methods: At a single tertiary care centre, patients undergoing primary TJA between July 2019 and January 2020 were identified using current procedural codes. Data were abstracted from the Institutional National Surgical Quality Improvement Program (NSQIP) along with electronic medical records which included referral type, primary insurance, demographics, comorbidities, and comorbidity scores, including an American Society of Anesthesiology (ASA) score. Complications and outcomes were tracked for 90 days post-operatively. Referral groups were compared using *Chi*-square exact tests for categorical variables and t-tests or Wilcoxon Rank Sum tests for continuous variables, as appropriate.

Results: Of the 393 patients included in this study, there were 249 (63%) NOR, 104 (26%) OOR, and 40 (10%) SR. The OOR versus NOR group had a significantly greater proportion of patients with obesity (79 vs 64%, p=0.047) and an ASA score ≥3 (59 vs 43%, p=0.007). There was a significantly greater proportion of patients with wound complications (10 vs 4%, p=0.023) and ≥2 complications (14 vs 3%, p<0.001) in OOR versus NOR, respectively.

Conclusion: Patients who underwent primary TJA and were referred by an orthopaedic surgeon tended to have more comorbid conditions and higher rates of severe complications. The observed difference in referrals may be explained by monetary incentivization in the context of current reimbursement trends. Organizations utilizing bundled payment programs to reimburse surgeons should use a risk-stratification model to mitigate incentivizing surgeons to avoid medically complex patients.

## Introduction

The U.S. spends more on health care than any other nation in the world at 19.7% of the gross domestic product (GDP) [1]. Health care spending nationwide is predicted to continue growing (5.4% per year). In particular, Medicare is expected to experience an even greater increase in spending (7.6% per year) given the aging baby boomer generation [1].

Primary total hip and knee arthroplasty (THA and TKA, respectively) are two of the most common and expensive orthopaedic procedures paid for by the Centers for Medicare and Medicaid Services (CMS) and they are expected to double by 2060 [2-4]. Thus, an early focus on value-based total joint arthroplasty (TJA) was made by CMS with the first installment being Bundled Payment for Care Improvement (BPCI) enacted in 2013 [5]. This created a single bundled payment for all the services provided to a patient during a TJA and any associated services up to 90 days post-operatively. Subsequent installments by CMS include the Comprehensive Care for Joint Replacement (CJR) Model and most recently, the BPCI-Advanced (BPCI-A) [5,6]. Commercial payors have been much more reluctant to follow CMS’s lead. It is estimated that less than 2% of commercial payments are processed through bundled payments [7].

Orthopaedic surgeons and hospitals have embraced these policies by working together with the common goal of reducing costs, improving outcomes, and minimizing complications. In this respect, they have been quite successful [7-12]. Despite the proven cost-effectiveness of TJA, surgeon reimbursement and margins have been steadily declining, with recent reporting of negative margins at some institutions since BPCI-A was enacted in 2018 [5,13,14]. Surgeons are often not adequality compensated for managing sicker patients [15]. This has led to a growing concern that orthopaedic surgeons are being incentivized to avoid medically complex patients.

The purpose of this study was to determine if there was a difference in comorbidities, demographics, or outcomes for patients undergoing primary THA and TKA based on referral type: non-orthopaedic referral (NOR), outside orthopaedic referral (OOR), and self-referral (SR). We hypothesized that OOR compared to NOR patients would be more medically complex and have higher rates of complications given the trend in TJA reimbursement and prior literature showing that tertiary care centres are increasingly being burdened with more comorbid patients [5,11,13-22].

This article was presented as a poster presentation at the Orthopedic Research Society 2024 Annual Meeting on February 4, 2024, and at the 2024 Medical Student Research Conference at the University of Iowa on September 13, 2023.

## Materials and methods

Data collection

This retrospective study was approved by our local institutional review board (IRB). Patients who underwent primary THA and TKA from July 2019 to January 2020 were identified using current procedural terminology (CPT) codes 27130 and 27447, respectively. Patients without at least 90-day follow-up and/or no referral on record were excluded. Out of 502 patients, a total of 109 patients were excluded because of at least one of the two aforementioned exclusion criteria. Data were abstracted from the institutional National Surgical Quality Improvement Program (NSQIP) and electronic medical records (EMRs). Data abstracted included referral type (recorded at the initial visit for procedure), primary insurance, demographic information, body mass index (BMI), smoking status in the past year, diabetes, Charleston Comorbidity Index (CCI), and American Society of Anesthesiology (ASA) score. The CCI is a validated comorbidity index to predict 10-year survival [23]. The referral type was determined by an internet search of referring physicians found in the EMR.

Complications following TJA were collected for the 90-day post-operative period and included perioperative fracture, transfusions, surgical site infection, wound complications, unplanned antibiotics (defined as antibiotics that were prescribed post-operatively and not a part of the pre-operative plan, i.e. prophylactic antibiotics given to patients with high-risk status for infection were not included in this group), prosthetic joint infection (PJI), knee instability, hip dislocation, pulmonary embolism/deep vein thrombosis, cardiovascular adverse event, length of stay (LOS), procedural length, reoperations, number of admissions within 90 days postoperatively, and total number of complications. Patients were categorized based on the referral source as (i) NOR, (ii) OOR or (iii) SR. NOR includes referrals from any providers who were not orthopaedic surgeons, predominately primary care providers. OOR consisted of referrals that were from orthopaedic surgeons outside of our institution. SR consisted of referrals that were from the patient themself.

Data analysis

Categorical variables were compared between groups using Chi-square or Fisher’s exact test as appropriate. Logistic regression was used to compare the odds of complications between referral groups using the OOR group as the reference. Between group differences in continuous variables were tested using generalized linear models. If results were statistically significant, this was followed by comparisons between NOR and OOR as well as SR and OOR using independent t-tests. Between group differences in continuous variables with non-normal distributions were tested using Kruskal-Wallis tests, and if statistically significant, followed by Wilcoxon Rank Sum tests. P-values were adjusted for multiple comparisons using the Stepdown Bonferroni approach.

A sample size estimate was completed using the formula, N =p(1-p) (Z/E)^2^ [24]. A power of 80% was utilized, the margin of error was estimated at 0.1, the proportion was assumed at 0.5, and a significance of 0.05 was utilized to avoid type II and I errors. With dichotomous outcomes, the analysis showed that approximately 100 patients would be required in each arm; however, we increased the aim to a total of 400 patients given that there was a lack of prior literature and an unknown rate of referral.

## Results

Of the 393 patients included, 221 (60%) underwent TKA and 172 underwent THA (40%) (Table 1). Of the 393 patients, 165 (42%) of patients were covered by Medicare and the average age was 61.8 ± 12.2 years old, 52% of patients were female, and 363 (93%) were white. There were 249 (63%) NOR, 104 (26%) OOR, and 40 (10%) SR (Table 2).

**Table 1:** Prevalence of total hip and knee arthroplasty breakdown by the referral group Table showing the proportions and prevalence of total hip (THA) and knee arthroplasty (TKA) between the referral cohorts (P-value = 0.1084). Non-orthopaedic referral (NOR). Outside orthopaedic referral (OOR). Self-referral (SR).

Referral Group	THA	TKA
NOR	99 (40%)	150 (60%)
OOR	53 (51%)	51 (49%)
SR	20 (50%)	20 (50%)

**Table 2:** Referral groups' demographics and comorbidities Table showing the prevalence of all the patient characteristics broken down into different referral cohorts: non-orthopaedic referral (NOR), outside orthopaedic referral (OOR), and self-referral (SR). Interquartile range (IQR). Standard deviation (std). American Society of Anesthesiology (ASA). Charleston Comorbidity Index (CCI). *Indicates statistic is given as an F-value.

Measure	Referral Groups			P-value	Test Statistic
	NOR (reference=1) n=249	OOR (reference=2) n=104	SR (reference=3) n=40		Chi-square
Age (mean, ±std)	62.6±11.7	60.2±13.4	60.9±12.1	0.2176	1.53*
Race				0.9799	1.2519
White	229 (92%)	98 (94%)	38 (95%)		
Black	15 (6%)	5 (5%)	2 (5%)		
Other	4 (2%)	1 (1%)	0		
Sex (n, % female)	134 (54%)	50 (48%)	22 (55%)	0.5805	1.0876
Medicare (n, % yes)	102 (43%)	50 (51%)	13 (34%)	0.1763	3.4716
Medicaid (n, % yes)	17 (7%)	6 (6%)	5 (13%)	0.3555	2.0687
Obese (n, % BMI ≥30)	158 (64%)	82 (79%)	22 (55%)	0.0051	10.5489
				1 v 2: 0.0084	7.9860
				3 v 2: 0.0084	8.1884
Morbidly Obese (n, % ≥40)	45 (18%)	27 (26%)	7 (18%)	0.2197	3.0306
Smoker (n, % past year)	13 (6%)	4 (4%)	2 (6%)	0.8672	0.2849
Diabetes	40 (17%)	14 (15%)	2 (6%)	0.1925	3.2948
High ASA (n, % ≥3)	107 (43%)	61 (59%)	14 (35%)	0.0085	9.5471
				1 v 2: 0.0072	7.2331
				3 v 2: 0.0109	6.4766
High CCI (n, % ≥3)	131 (53%)	49 (47%)	13 (33%)	0.0550	5.8020

Between-group differences in comorbid conditions

The OOR versus NOR group had a significantly greater proportion of patients with obesity and a high ASA score, defined as an ASA score ≥3, but a similar proportion of female and Medicare patients (Table 2). Morbid obesity was higher for OOR compared to NOR but not significantly so. Interestingly, a greater proportion with a high CCI (score ≥3) was noted in NOR versus OOR, but this did not achieve statistical significance (Table 2). The CCI is a validated comorbidity index to predict 10-year survival [23]. The OOR versus SR group had significantly higher rates of obesity and high ASA score (Table 2). There were no significant differences between groups in smoking and diabetes status (Table 2).

Between-group differences in post-operative outcomes

The OOR group compared with the NOR group had significantly higher rates of patients with ≥2 complications, wound complications, unplanned antibiotics, blood transfusion, and longer median surgery duration (Table 3). There was significantly lower odds of post-operative wound complication and 2 or more complications for NOR vs OOR, respectively (Table 4). Continuing the trend NOR patients had lower odds of readmission and any complication when compared to the OOR patients, but never obtained significance (Table 4). While higher proportions of OOR versus NOR patients returned to the operating room (OR) (5 vs 1%), experienced readmission (4 vs 3%), and experienced any form of complication (19 vs 12%), these differences were not significant (Table 3, all P > 0.05). Patients had a significantly longer surgery length and median length of stay in OOR versus SR group. (Table 3). Interestingly, the SR group had similar prevalence of wound complications and unplanned antibiotics when compared to the OOR group (Table 3). There were lower odds of two or more complications, readmission and any complication for the SR group versus OOR group, but not enough to obtain significance (Table 4; P>0.05). 

**Table 3:** Complications of referral groups Prevalence of all the complications variables recorded divided by referral group: non-orthopaedic referral (NOR), outside orthopaedic referral (OOR), and self-referral (SR). Interquartile range (IQR). Periprosthetic joint infection (PJI). Superficial soft tissue infection (SSI).

Measure	Referral Groups			P-value	Test Statistic
	NOR (reference=1)	OOR (reference=2)	SR (reference=3)		Chi-square
Reoperation rate (n, % yes)	3 (1%)	5 (5%)	1 (3%)	0.1331	4.2644
Readmission (n, % yes)	7 (3%)	4 (4%)	1 (3%)	0.9004	0.3115
Wound complication (n, % yes)	9 (4%)	10 (10%)	4 (10%)	0.0454	6.1849
				1 v 2: 0.0228	5.1873
				3 v 2: 1.0000	0.0049
SSI (n, % yes)	2 (0.8%)	2 (2%)	0	0.7299	1.3711
Deep wound complication (n, % yes)	0	0	0	-	-
Sepsis (n, % yes)	1 (0.4%)	0	1 (3%)	0.2630	3.7206
Blood transfusion (n, % yes)	0	3 (3%)	0	0.0272	8.4007
				1 v 2: 0.0251	7.2443
				3 v 2: 0.5604	1.1784
Any complication (n, % yes)	29 (12%)	20 (19%)	6 (15%)	0.1598	3.5431
Total number of complications (n, % ≥2)	8 (3%)	14 (14%)	2 (5%)	0.0012	13.5334
				1 v 2: 0.0003	13.1854
				3 v 2: 0.1479	2.0942
Intra-peri operative fracture (n, % yes)	3 (1%)	1 (1%)	0	1.0000	0.5010
Unplanned antibiotic use (n, % yes)	12 (5%)	12 (12%)	4 (10%)	0.0455	5.7385
				1 v 2: 0.0207	5.3517
				3 v 2: 1.0000	0.0550
PJI (n, % yes)	3 (1%)	5 (5%)	1 (3%)	0.1338	4.2369
Dislocation (n, % yes)	3 (1%)	2 (2%)	0	0.7838	0.8820
DVT/PE (n, % yes)	1 (0.4%)	1 (1%)	0	0.5974	0.6793
Cardiac complication (n, % yes)	3 (1%)	2 (2%)	0	0.7802	0.8594
Length of stay (LOS) (median, IQR)	1 (1-2)	1 (1-2)	1(1-1)	0.0126	8.7420
				1 v 2: 0.0551	3.6804
				3 v 2: 0.0126	7.4757
Procedural length (median, IQR)	100 (89-114)	106.5 (89-139)	93 (81-112)	0.0219	7.6433
				1 v 2: 0.0404	4.3352
				3 v 2: 0.0404	5.4089
Discharge Disposition Home (n, % yes)	232 (93%)	91 (88%)	38 (95%)	0.1538	3.7442

**Table 4:** Calculated odds ratios for complications Calculated odds ratios (OR) for wound complication, two or more total complications, readmission rate with corresponding 95% confidence interval (CI) and p-value. Non-orthopaedic referral (NOR). Outside orthopaedic referral (OOR). Self-referral (SR).

Measure	OR (95%CI)	P-value
Wound Complication		
NOR v OOR:	0.352 (0.139-0.895)	0.0282
SR vs OOR:	1.044 (0.308-3.543)	0.9443
Two or more total complications		
NOR v OOR:	0.213 (0.087-0.526)	0.0008
SR vs OOR:	0.338 (0.073-1.562)	0.1649
Readmission		
NOR v OOR:	0.723 (0.207-2.525)	0.6114
SR vs OOR:	0.641 (0.069-5.916)	0.6949
Any Complication		
NOR v OOR:	0.554 (0.297-1.032)	0.0627
SR vs OOR:	0.741 (0.274-2.006)	0.5554

## Discussion

The purpose of the study was to determine if there was a difference in comorbidities and outcomes in patients undergoing primary THA and TKA based on the referral type. This study found patients referred by an outside orthopaedic surgeon had more comorbid conditions. Specifically, a greater proportion of these patients were obese and had high ASA scores. The ASA score is a validated scoring system meant to estimate a patient’s perioperative risk [25]. However, the insurance type did not seem to play a role (Table 2). There are multiple possible explanations for this trend, and it is likely that a component of each is at play.

Tertiary care centres are meant to treat medically complex patients, therefore orthopaedic surgeons could be referring patients because their facilities lack the resources or means to provide the level of care necessary for medically complex patients. Tertiary care centres then would offer optimization and specialisation to minimise patient complications and improve outcomes for the appropriate patient [9,26].

Alternatively, given the surgeon reimbursement trends, orthopaedic surgeons could be letting monetary risks influence their decision to refer patients despite being capable of adequately treating the patient [13-15,17]. Evidence to support this conclusion can be seen in a study by Haglin et al., which demonstrated that arthroplasty surgeons were receiving lower mean reimbursement for sicker patients [15]. CMS target pricing methods have led to an inflation-adjusted 1.7% annual decline in reimbursement for arthroplasty and, combined with reports of negative margins since 2019 from BPCI-A, it is reasonable to believe that monetary incentives could be contributing to the decision to refer [13,14]. If this is the case, it would be expected that orthopaedic referrals would be disproportionately covered by Medicare. In this study, while the OOR group had a higher proportion of patients with Medicare coverage, it was not significant. While the study was powered to 80%, that does not eliminate the chance this study was underpowered to recognise the underlying difference and thus warrants further investigation.

While tertiary care centres are meant to deal with sicker patients and complex pathology, recent literature has shown that these facilities are being increasingly burdened [19,22,27]. Additionally, high-volume centres have been shown to decrease complications for the right patients but simultaneously inappropriate referrals have been shown to cause access-of-care issues and exacerbate complications [22,26]. Not all comorbidities necessitate tertiary care or high-volume centres and thus require proper referral. For instance, local arthroplasty surgeons should be capable of treating patients with obesity.

Compared to the NOR group, the OOR group of patients had significantly higher rates of patients with two or more complications, wound complications, unplanned antibiotics, blood transfusions and longer surgery length. Longer surgery length for OOR patients could indicate local orthopaedist preference to operate on more routine patients. Admittedly, with only a six and half minute difference in operation time, this observation is of questionable clinical significance. The more comorbid patients in the OOR group are likely contributing to the increased complications. Access to care could be exacerbating complications in these patients and could explain why they have more severe complications. In orthopaedics, geographic and financial barriers to care have been proven to play a significant role in complications by having greater travelling distances as well as being financially disadvantaged [21,22]. Rural populations, which make up a sizable portion of the population around our tertiary centre, are particularly vulnerable to access to care issues, [21].

In this study, higher rates of multiple complications were observed in the OOR group (readmissions, reoperations, any complication, etc.) compared to NOR and SR, but these trends did not reach statistical significance (Table 3). While many of the individual variables did not reach significance, the trend is not insignificant and should not be ignored, as it may indicate differences that would be significant in a greater sample size. Such large-scale studies could be facilitated if quality improvement programs like NSQIP or CMS were to track referrals.

Despite the proven clinical and cost benefits of THA and TKA, CMS continue to target pricing methods that reduce profit margins for arthroplasty surgeons [13,14,28]. This study evaluated patients before CMS released their 2021 Physician Fee Schedule, in which they further decreased the reimbursement for TJA [29]. This is likely to increase the probability of arthroplasty surgeons avoiding more comorbid patients to ensure positive margins. We recommend that bundled payment reimbursement programs for TJA, like that of CMS, start a risk-stratified reimbursement system for orthopaedic surgeons to avoid cherry-picking patients and ensure equitable access to care for patients.

In our study, 27% of patients undergoing primary TJA at our tertiary care centre were referred by local outside orthopaedic surgeons. To the best of the author’s knowledge, this is the first report on the prevalence of orthopaedic referral rates to a tertiary care centre. This is just one institution’s findings and would benefit from investigation at other institutions as well as longitudinal tracking to observe referral trends as reimbursement continues to evolve.

One limitation of this study is its retrospective design. Motivation behind referral is impossible to delineate retrospectively. Furthermore, there could be a component of selection bias given almost one-fifth of the patients selected need to be excluded. The study also did not evaluate patients who were referred and ultimately never underwent surgery. This could exclude the sickest patient population. By excluding this population, we did not include the sickest of the referred patients in our analysis. The inclusion of this population would likely have increased the observed differences in health between referral groups. Additionally, bundled payments currently only apply to the Medicare population and this study included all payors. Finally, the homogenous nature of the patient population limits this study’s generalizability. Finally, the homogenous nature of the patient population limits this study’s generalizability.

Future research is needed to determine if patients with multiple comorbidities are more likely to be referred when they have CMS coverage because CMS is the main user of bundled payments. This study did not evaluate travel distance and socioeconomic status and their relation to orthopaedic referrals. The findings are one institution’s findings and would benefit from a larger cohort from differing regions to increase generalizability.

## Conclusions

In conclusion, our tertiary care centre is being burdened with more comorbid patients from outside orthopaedic surgeons. The higher comorbidity in this patient population is likely contributing to an increase in the severity and rate of complications. Concurrently, given the surgeon reimbursement policy for primary TJA, the monetary incentive to refer more comorbid patients may be contributing to our institution's referral burden. In addition, this trend increases the risk of geographic access to care issues for an already vulnerable patient population, which could then further exacerbate complication rates. We urge organisations using bundled systems to implement a risk-stratified surgeon reimbursement strategy to avoid improper referrals and ensure equitable access to care for all patients.
